# Annotations

**Published:** 1893-12-16

**Authors:** 


### Annotations.

### A Little More Art.

The high scientific training carried out in the manufacture of practising doctors does not seem to be quite all gain when looked at from the point of view of the general community. It is not very encouraging to think that the life of a man like Professor Tyndall, or, indeed, the life of any other person who may happen to require the treatment of more than one symptom of unhealthiness at the same moment, may be sacrificed to what appears to have been the accident of an accident. Nothing can be easier than to prevent such calamities as that which overtook Mrs. Tyndall on the morning of her husband's death. Several suggestions are before the public, any one of which would effect the purpose. Such suggestions have been made and published many times before. But the real question is who, among the thirty-six millions of our population, is going to be responsible for the universal adoption of these suggestions? "Parliament!" Probably; but in order to get Parliament to move somebody must make himself, or some public organisation must make itself, responsible for the bringing in of a Bill. Who is to do this? The medical profession or the chemists ought certainly to put it forward. One of the easiest things in the world would be to make accidental poisoning by medicinal substances impossible. In our judgment, doctors, as the responsible custodians of the common health, should feel it their duty to bring the law up to the standard required by the public safety. If our scientific and learned profession cannot charge itself with the details of its own public work, so much the worse for its learning and science. Medicine, we insist, is as much responsible for the common health as Parliament is for the common politics. We must charge ourselves with details; and not only with the details of our individual patients' safety, but with the details of drug administration as they affect the public safety. The College of Physicians, or even the British Medical Association, should bring this disgraceful state of things to a termination, unless, indeed, the Medical Department of the Local Government Board can promptly undertake the duty.

### The Carriage of the Dead by Railways.

One of the small reforms demanded by the common sentiments of humanity is the power to transmit dead bodies to a distance at reasonable charges. We publish elsewhere the story of the last illness and death of a Bedford labourer, named Partridge. Partridge, it would appear, was dangerously ill, and the Bedford Guardians, wishing to give him every possible chance of life, offered to pay his expenses to and from London, if he would consent to submit his case to the doctors of St. George's Hospital. After much persuasion the poor man consented; but his feeble faith was not rewarded, and he died in the hospital. The Guardians had paid 4s. 7d. for the transmission of the living body; but when it came to be a question of bringing the dead body back again for burial among his friends, not 4s. 7d, but 57s., or more than twelve times the amount of a living person's fare was the sum demanded. This the Guardians hesitated to pay, though they compromised the matter by paying 50s. for the poor man's coffin, whilst a benevolent lady, Miss Mary Thomas, paid the 57s. demanded by the railway company for the carriage of the body from London to Bedford. There may be some reason peculiar to railway companies why they should charge such extremely exorbitant sums for the transmission of dead human bodies, but we certainly cannot imagine what it may be. The carcases of dead animals are not charged at these rates, or indeed at abnormal rates at all. Before denouncing we should like to hear what the railway companies have to say on the subject, for they all seem to act by the same rules. If there be no reason, as we suspect there is not, beyond a mere determination to avoid carrying human corpses by the charging of prohibitory rates, then we think the companies make a grave mistake, and fail in a just appreciation of public duty. The question is certainly one which demands the consideration of Parliament, and also of the public and the press.

### Beef, Butchers, and Buyers.

A great deal of the "roast beef of old England" will be bought next week for Christmas consumption. It is worth while now that "beef" is in the air, to devote a moment's consideration to this same "fine English roast beef." The report of the Select Committee of the House of Lords on the marking of foreign meat has just been published, and to the man who believes in paying no more than a shilling for a market shilling's-worth of goods, it is most exasperating reading. If the evidence of the witnesses can be relied on, it is quite certain that English butchers, as a class, have sunk to the very lowest possible ebb in elementary morals. Here, for instance, is a story which is a fair sample of the evidence given with regard to beef, bacon, margarine, cheese, butter, and every variety of foreign produce which can be manipulated by plausible rogues and sold for English prices in the English market: A woman went into a butcher's shop at a seaside town not long ago and asked to be supplied with some American meat because it was cheaper. "I have never kept American meat in my shop," said the virtuous British butcher; "I should not think of keeping it." "Well," said the woman, "that is a very funny thing, because my husband is one of the salesmen in the Smithfield Meat Market, and sells you American meat, and he told me when I came down here to go and deal with you, because you were such an excellent customer to him." Several witnesses, such as butcher's clerks, assistants, and retired butchers swore that in their experience three-fourths of the meat sold as English, and at English prices, is American. Now the price of American meat to the butcher is not more than two-thirds the price of English. Of course there is no objection to the sale of American meat, so long as it is sold as American and at American prices. But to sell it as English, and at English prices, is a vile fraud on the part of the butcher, a ruinous injury to English agriculture, and a robbing of the customer. We put it to our fellow-countrymen that we must be a set of helpless noodles when we permit ourselves to be swindled daily with our eyes open in this fashion. The County Councils should take such questions up. What is the use of them if they do not? Of course the butchers ought to be ashamed to be such rogues. But it is all too evident that roguery which is profitable and safe is an irresistible temptation to all but the very smallest fraction of the butcher class.

